# *Solanum torvum* responses to the root-knot nematode *Meloidogyne incognita*

**DOI:** 10.1186/1471-2164-14-540

**Published:** 2013-08-09

**Authors:** Paolo Bagnaresi, Tea Sala, Tiziana Irdani, Cristina Scotto, Antonella Lamontanara, Massimiliano Beretta, Giuseppe Leonardo Rotino, Sara Sestili, Luigi Cattivelli, Emidio Sabatini

**Affiliations:** 1 Consiglio per la Ricerca e la Sperimentazione in Agricoltura, Genomics Research Centre, via S Protaso 302, I-29107, Fiorenzuola d’Arda (PC), Italy; 2 Consiglio per la Ricerca e la Sperimentazione in Agricoltura, Unità di Ricerca per l’Orticoltura, Montanaso Lombardo (LO), Italy; 3 Consiglio per la Ricerca e la Sperimentazione in Agricoltura, Centro di ricerca per l’agrobiologia e la pedologia, Cascine del Riccio, 50125, Firenze, Italy; 4 UNIMORE, Scienze Agrarie e degli Alimenti, Università degli Studi di Modena e Reggio Emilia, via Giovanni Amendola 2, Padiglione Besta, Reggio Emilia 42122, Italy; 5 Consiglio per la Ricerca e la Sperimentazione in Agricoltura, Unità di Ricerca per l’Orticoltura, Monsampolo del Tronto, AP, Italy

**Keywords:** Torvum, Nematode resistance, 454 pyrosequencing, Microarray, Heterologous hybridizations

## Abstract

**Background:**

*Solanum torvum* Sw is worldwide employed as rootstock for eggplant cultivation because of its vigour and resistance/tolerance to the most serious soil-borne diseases as bacterial, fungal wilts and root-knot nematodes. The little information on *Solanum torvum* (hereafter Torvum) resistance mechanisms, is mostly attributable to the lack of genomic tools (e.g. dedicated microarray) as well as to the paucity of database information limiting high-throughput expression studies in Torvum.

**Results:**

As a first step towards transcriptome profiling of Torvum inoculated with the nematode *M. incognita*, we built a Torvum 3’ transcript catalogue. One-quarter of a 454 full run resulted in 205,591 quality-filtered reads. *De novo* assembly yielded 24,922 contigs and 11,875 singletons. Similarity searches of the *S. torvum* transcript tags catalogue produced 12,344 annotations. A 30,0000 features custom combimatrix chip was then designed and microarray hybridizations were conducted for both control and 14 dpi (day post inoculation) with *Meloidogyne incognita-*infected roots samples resulting in 390 differentially expressed genes (DEG). We also tested the chip with samples from the phylogenetically-related nematode-susceptible eggplant species *Solanum melongena*. An in-silico validation strategy was developed based on assessment of sequence similarity among Torvum probes and eggplant expressed sequences available in public repositories. GO term enrichment analyses with the 390 Torvum DEG revealed enhancement of several processes as chitin catabolism and sesquiterpenoids biosynthesis, while no GO term enrichment was found with eggplant DEG.

The genes identified from *S. torvum* catalogue, bearing high similarity to known nematode resistance genes, were further investigated in view of their potential role in the nematode resistance mechanism.

**Conclusions:**

By combining 454 pyrosequencing and microarray technology we were able to conduct a cost-effective global transcriptome profiling in a non-model species. In addition, the development of an *in silico* validation strategy allowed to further extend the use of the custom chip to a related species and to assess by comparison the expression of selected genes without major concerns of artifacts. The expression profiling of *S. torvum* responses to nematode infection points to sesquiterpenoids and chitinases as major effectors of nematode resistance*.* The availability of the long sequence tags in *S. torvum* catalogue will allow precise identification of active nematocide/nematostatic compounds and associated enzymes posing the basis for exploitation of these resistance mechanisms in other species.

## Background

*Solanum torvum* Sw (hereafter Torvum) is worldwide employed as rootstock for eggplant cultivation because of its vigor and resistance/tolerance to heavy metal- and salt-contaminated soil and, specially, to the most serious soil-borne diseases (i.e. bacterial, fungal wilts and root-knot nematodes) [1]. However, despite the fact that identification of the effectors of Torvum resistance would be extremely valuable for other solanaceous crops, particularly eggplant, very little studies have been conducted to date regarding Torvum resistance mechanism and have mainly dealt with its resistance to bacteria [2]. This scenario is mostly attributable to the paucity of database information on Torvum which, in turn is a consequence of the lack of high-throughput genomic tools (e.g. dedicated microarray SNP panels and genome sequencing)).

Few expression databases were available for Torvum. Only recently, 6,296 unigenes from Torvum cv. Torubamubiga, a low cadmium (Cd)-accumulating variety, have been obtained in the context of studies on Cd acclimation process [3]. This gene catalogue represents a significant improvement in terms of sequence information; however, given the current high-throughput standards, is unsuitable to pose the basis for global transcript profiling (e.g. to design probes for a comprehensive chip) due to its small size.

An approach to overcome such typical non-model species constraints would be undertaking an RNA-sequencing approach, since no prior knowledge of transcript sequences is mandatory. An alternative is represented by microarray hybridizations, a robust and well-assessed technique, about ten-fold less expensive per sample when compared to the cheapest RNA-Seq approaches [4]. Microarray can be considered as a viable alternative to RNA-Seq provided that the number of available sequences for the species of interest is considered satisfactory. Thus, in order to extend the number of probes for Torvum, we combined 454-based pyrosequencing with microarrays as reported by [5]. A further example of this strategy has been recently presented [6].

Root-knot nematodes, Meloidogyne spp. determine substantial losses on horticultural and field crops [7]. In the tropical and sub-tropical regions, crop production losses due to nematodes were estimated at 14.6% compared with 8.8% in developed countries [8]. The American Phytopathological Society has reported that root-knot nematodes are the most common and destructive nematode pathogens, accounting for an estimated of 14% of all worldwide plant losses, which translates into almost $ 100 billion dollars annually [9].

*Meloidogyne incognita* belongs to the order Tylenchida, a very large and diverse group of nematodes, which contains a majority of the known plant parasitic species. Representatives of this order have a worldwide distribution and are encountered more frequently than any other group of nematodes. Within Tylenchida, members of the family Heteroderidae are by far the most damaging to world agriculture, among which the root knot nematodes (RKN) (Meloidogyne spp.) and the cyst nematodes (Globodera spp. and Heterodera spp). To date, more than 80 RKN species are described, and M. incognita is unquestionably the most important one in terms of distribution and damages. Nematodes establish complex interactions with hosts, and, upon successful infection, cause the reprogramming of host cell developing structures as multinucleate giant feeding cells. More than 400 proteins have been found to be secreted by M. incognita [10] and several of these proteins are thought to play a role in modulating infection and reprogramming host metabolism.

Nematode Resistant (Nem-R) genes are an obvious topic of interest for crop protection. The first cloned Nem-R gene was the sugar beet Hs1pro-1 conferring resistance against the sugar beet cyst nematode [11]. However, Hs1pro-1 appears poorly related to typical plant R genes. Several other Nem-R genes (e.g. Mi-1, Hero A, Gpa2 and Gro1-4, all cloned from tomato or potato relatives) belong to the NBS-LRR class of R-genes [12]. With respect to the subclass of RKN R-genes (RKN-R), few resistance genes have been identified and only two have been cloned, namely Mi-1 from tomato [13] and, very recently, Ma from the Myrobalan plum Prunus cerasifera [14]. Both Mi-1 and Ma confer broad-spectrum resistance against several root knot nematode. Not only a single pathogen-related gene in response to nematodes attack is described in the literature. More than one major gene is often involved in the resistance mechanisms commonly referred as horizontal resistance.

Wang *et al.*[15] identified one major recessive gene with strong additive effect against *M. incognita* in cotton, together with a major QTL with strong dominant effect in different crosses using different resistance sources. Transgressive segregation was previously documented from the same authors [16] as an epistatic interaction that strengthened the resistance phenotype. The determinants of Torvum resistance to nematodes are yet to be identified, and, in particular, the contribution of a specific gene-for-gene interaction rather than an horizontal, quantitative resistance, or even a “non-host” resistance phenomena (or a combination of these mechanisms) is far from being clear.

A number of studies have investigated transcriptional changes of *M. incognita*-challenged compatible and incompatible plants in a variety of species. Two early studies described the transcript profiling of about 900 transporter genes in *A thaliana*[17] and of 1,547 selected genes in tomato [18]. The latter work was carried out on resistant and susceptible tomato genotypes and, by comparing transcript abundance patterns over a time-course, several clusters of genes associated to compatible and incompatible interactions were accurately defined. Furthermore, a glycosyltransferase was shown to be necessary for conferring the resistant phenotype [18].

A larger number of genes (22,089) was tested by Jammes et al. [19] in Arabidopsis, using giant cell-enriched root tissues infected by *M. incognita* along three time-points. About 3,000 genes were called as differentially expressed between uninfected root tissues and galls at different developmental stages. Major classes of modulated genes included metabolism-associated genes and transcription factors. A substantial down-regulation of defense genes, including phenylpropanoids, was found.

More recently [20], the expression profiling of about 2,000 genes from EST libraries obtained from *M. incognita*-challenged resistant and susceptible cotton varieties, revealed differential modulation in resistant and susceptible genotypes of genes falling in the broad categories of pathogen recognition, signal transduction and pathogen defense. This latter category included superoxide dismutases, chitinases and isoflavone reductases. A further recent work [21] have described the changes in the soybean transcriptome using the Affymetrix^®^ Soybean Genome Array (37,500 genes) in galls formed in *M. incognita*-challenged roots at 12 days and 10 weeks after infection. Genes encoding enzymes involved in carbohydrate and cell wall metabolism, cell-cycle control and plant defense were significantly modulated. Among plant defense genes, several lipoxygenases, pathogenesis-related proteins and phenylpropanoid genes were found to be differentially expressed.

The aim of this work was, by global transcriptome profiling, getting insights on the mechanisms underpinning Torvum resistance against *M. incognita*. Toward this end, we deployed an approach which takes advantage of both Next generation sequencing (NGS) and microarray techniques. In particular, we targeted previously uncharacterized transcripts by RNA-Seq and exploited reliability and cost-effectiveness of well established microarray technologies for transcript quantification. We also explored the “chip extension” procedure, an approach allowing to increase the reliability of heterologous hybridizations by defining subset of probes less likely to be prone to expression artifacts. The availability of a 3′ transcript catalogue for Torvum and transcript profiling upon nematode infection provides molecular tools for identifying Torvum resistance mechanism.

## Results and discussion

### De novo assembly

Since only 6,296 unigenes from Torvum are available to date [3], we undertook an RNA-Seq pyrosequencing approach (454-GS-FLX technology with Titanium chemistry) to extend the number of Torvum genes and thus pose the basis to perform a global transcriptome profiling in a cost-effective manner. According to [5] we produced a comprehensive normalized catalogue of the 3’ mRNA regions (500–600 bp) tailored at the generation of a custom chip. As starting material, Torvum roots were subjected to a wide range of environmental stresses (see Materials and Methods) to maximize the number of expressed genes. Sequencing was confined to the 3’ in order to: i) minimize the number of 454 reads mapping to the same transcript but assembling in different contigs due to the lack of uniform coverage in low-abundance transcripts (e.g. transcripts where reads do map towards 5’ and 3’ regions but not in intervening region of the same RNA molecule); ii) allow for designing highly specific probes by encompassing 3’ regions which are known to be subjected to lower selection pressure. The library was normalized as this treatment has been shown to greatly improve rare transcript coverage as well as other quality features [5]. One-quarter of the 454 plate was used to sequence the normalized 3’cDNA library yielding 205,591 reads with an average length of 356 bases for a total of (trimmed and quality-filtered) 73,266,807 bases.

Assuming an average number of transcripts of 30,000 with an average length of 2 Kb and thus a transcriptome length of 60 Mb, an yield of 73 Mb confined to 500–600 bp at the 3’ regions represents a coverage of about 4x. This coverage, while not exhaustive, poses the basis, at least within the context of a normalized library, for quantification of a high number of transcripts in comparison to the few thousands of unigenes to date available for Torvum.

*De novo* assembly of Torvum reads was undertaken with MIRA 3.0.5 [22]. The assembly led to 24,922 contigs plus 11,875 singletons (Table 1). Various parameters including N50 were calculated to describe the average size of the contigs. In Additional file 1, the distribution of contig length (panel a) and contig coverage (panel b) is shown. As a consequence of our 3’ sequencing design, the most enriched bin for unigenes (contigs plus singletons) was, as expected, in the 500–600 bp region. Contig coverage (average number of reads per contig) was relatively uniform as a result of the normalization step [5].

**Table 1:** Summary of Torvum assembly

**Total reads**	**205.591**
N° of reads assembled	161.636
N° of singlets	11.875
Contigs	24.922
Total unigenes (contigs plus singletons)	36.797
Discarded reads	43.955
N10 contig size	715
N25 contig size	627
N50 contig size	514
N90 contig size	386
N95 contig size	327

To further assess the assembly, we compared the contigs plus singletons (hereafter referred to as Torvum unigenes) against selected public assemblies, including the recently released 6,296 unigene catalogue from *Solanum torvum* cv. Torubamubiga [3]. Further queried databases (as detailed in Materials and Methods) were the current releases in TC database from the phylogenetically related species eggplant (25,443 unigenes), tobacco (116,964), tomato (52,502), potato (62,330) and pepper (32,399). At last, we tested Arabidopsis (112,827 unigenes) as a phylogenetically distant reference. As expected, a limited number of Torvum queries showed hits against the small (6,296 unigenes) Torvum Torubamubiga dataset (about 40% of queries at E10^-6^), while the larger TC solanaceous datasets as potato, tomato, eggplant and tobacco exhibited between 70 and 80% hits. However, when these results are corrected for the number of entries of the queried databases (percent hits divided by number of entries in queried database), eggplant and *S. Torvum* cv. Torubamubiga clearly emerged as the most correlated to Torvum database. On the other hand, the phylogenetically distant species Arabidopsis shows a barely detectable ratio of percent hits to database extent (data not shown).

Overall, the blast data closely mirror known phylogenetic relationships within solanaceous species [23,24] with Torvum having its closest counterpart in eggplant and, in order of decreasing relatedness, potato, tomato, pepper and tobacco. Noteworthy, at an Expect value of 10^-6^, more than 60% of Torvum unigenes had no hits against cv. Torubamubiga database, indicating that a majority of Torvum unigenes in our catalogue are not represented in the small Torubamubiga dataset (6,296 unigenes). On the other hand, when Torubamubiga database was queried against our Torvum unigene catalogue, only 18% of the 6,296 Torubamubiga unigenes had no hits, indicating that our Torvum transcript tags catalogue is likely to represent the most complete dataset for Torvum available to date.

### Custom chip design

OligoArray 2.1 software [25] was used to compute gene-specific oligonucleotides corresponding to Torvum unigenes. OligoArray output, besides microarray design, provides hints on the quality (e.g. degree of redundancy) of input sequences by declaring how many specific probes can be designed based on input sequences. About 80% of oligos (30,162 out of 36,797 unigenes) turned out to be specific for one Torvum unigene, while 15% oligos were specific for 1–3 unigenes, (Additional file 2) indicating efficient normalization and substantial lack of redundancy in the Torvum unigene set. A final filtering step over Torvum unigenes was conducted to exclude the less specific probes (i.e., those corresponding to unigenes for which oligoarray could not design a strictly specific probe and would thus hybridize with more than one target). This also allowed to contain the number of probes in the chip to maximum 30,000, consistent with a triplicate probe layout in the 90k-features Combimatrix chip design. The final layout consisted in 24,394 probes representative of contigs and 5,606 probes derived from singletons. The significant reduction (more than 50%) of singleton representation in the final chip layout likely reflects the constraints experienced by oligoarray software in designing specific probes for singletons due to the shorter length and/or lower qualities of sequences when compared to contigs.

### Differentially expressed genes in nematode-infected Torvum

Two-months-old Torvum plants were infected with *Meloidogyne incognita* and left to proceed for 14 days. Neither root-galls (RG) nor egg-masses (EM) were never collected on Torvum stained root (RG-index = 0, EM = 0) even in longer infection stages (60 dpi).

**Figure 1:**
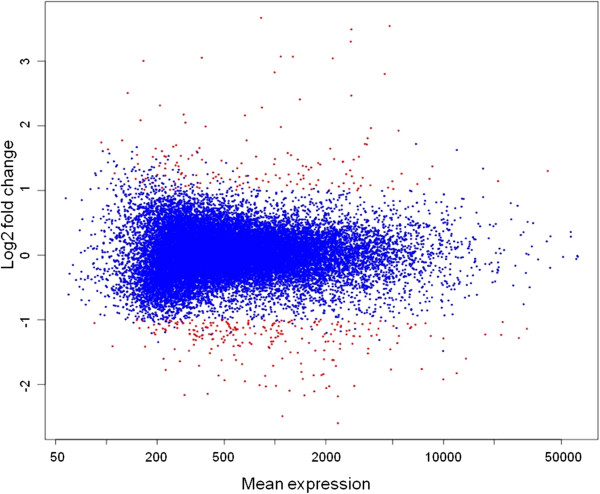
**Overview of Torvum transcriptional changes upon nematode infection.** The MA-plot represents mean expression values plotted *versus* log2 fold changes. Called DEGs (fold change > 2 and FDR < 0.1) are plotted in red.

Torvum RNA samples (three biological replicates for both control and inoculated roots) were used for array hybridizations. Pearson correlation coefficients for biological replicates were all above 0,85 (Additional file 3). Genes were considered differentially expressed if exhibiting at least a 2-fold change and a False Discovery Rate (FDR) <= 0.1. Figure 1 depicts transcript abundance values in control *vs.* infected roots as MA-plot. Differentially expressed genes (DEG) fulfilling the two criteria (390 genes) are plotted in red. The full list of DEG accompanied by expression ratio, FDR values, blast hits, Blast2GO annotations and GO mappings are reported in Additional file 4.

### Annotation of Torvum gene catalogue

The software suite Blast2GO [26] was used to annotate Torvum unigenes. As a first blast step, we run BlastX against NCBI non-redundant database using as query the 23,284 unigenes included in the chip layout for which an hybridization signal could be obtained. A total of 71,474 GO annotations were retrieved. The full catalogue of Torvum genes including Blast2GO annotations and complementary information (i.e. blast hit description for transcripts below Blast2GO annotation threshold) are available in Additional file 5, while the full set of Torvum unigene sequences (ID of contigs plus singletons and transcript sequences) is provided in fasta format in Additional file 6.

### Go enrichment analysis of torvum DEG genes

**Figure 2:**
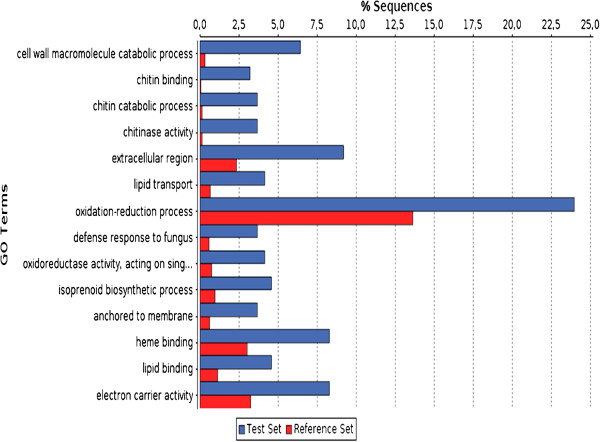
**GO term enrichment.** The most specific GO terms enriched in Torvum as a consequence of infection are shown. For each GO term, the percentages of Torvum unigenes in test set (DEG) or reference set (total annotated unigenes) are reported.

Major expression trends in infected Torvum were investigated to test whether the 390 DEGs could be grouped in some enriched GO terms. Towards this end, we used as a reference set the 12,344 annotations obtained with Blast2GO and as test set the 390 DEGs. The Fisher’s exact test results (FDR <= 0.1) for the entire set of enriched GO terms and corresponding Torvum unigenes is reported in Additional file 7. In Figure 2, the most specific enriched terms are combined in a chart. Among enriched GO terms, several fall within a generalized biotic stress, namely “cell wall macromolecule metabolic processes”, “extracellular region”, “response to stress”, “polysaccharide catabolic process” and “response to fungus”. Further enriched GO terms, including chitin and isoprenoid –associated processes appear of special interest within the context of nematode infection and will be analyzed in more detail.

### Chip-extension to eggplant

While the focus of this study was to investigate Torvum defense responses upon nematode infection, we reasoned that the custom chip designed in this work may as well provide valuable complementary information concerning eggplant, a phylogenetically close, nematode-susceptible species. Eggplants are most seriously affected by *Meloidogyne*, when plants of the breeding line 1F5(9) were infected with root knot nematodes a very high susceptibility with RG-index = 5 and EM = 5 was scored. To our knowledge, no genome-wide expression studies have been published to date for nematode-challenged eggplant.

Heterologous hybridizations are based on interrogation of a chip specifically designed for a species (primary species) with transcripts from a phylogenetically-related species (secondary species). Heterologous hybridizations have in many cases shown to be valuable resources for non-model species, especially when the primary and secondary species are closely related ([27]–[29] and references therein). Furthermore, in recent years, validation procedures have been developed allowing the definition in the secondary species of subsets of probes where expression data are predicted to be highly reliable [27]. These procedures exploit the fact that the probes designed for a primary species (i.e. generally perfectly matching to primary species transcripts) can be tested for matching to database transcripts of a secondary, interrogating species. This allows assessment of expression data reliability and eventually definition of subsets of genes in the secondary species which can be evaluated with reduced concerns of expression artifacts.

**Figure 3:**
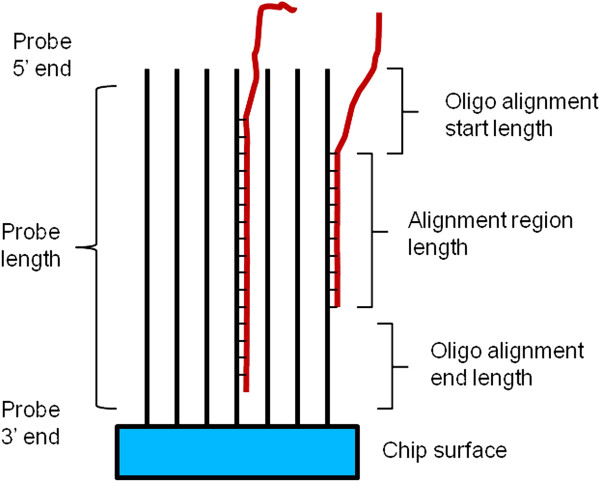
**Explanation of probe filtering metrics for eggplant transcripts.** Parameters used for filtering of Torvum probes based on homology to eggplant transcript are shown. Due to the steric constraints in the crowded 3’ attachment to chip region, the 3′ region of probes is less accessible and therefore mismatches in the region are less influential.

To validate the heterologous expression data, we followed an approach conceptually similar to that presented in Bagnaresi et al. [27]. Toward this end, we first pooled several expression (EST and assembled unigenes) eggplant databases and queried the merged eggplant database with Torvum chip probe sequences using local BlastN at a relaxed stringency. Alignment results were parsed to filter probes based on alignment parameters expected to influence hybridization strength. The following parameters were considered (see explanatory Figure 3): i) ratio of alignment length to oligo length (percent alignment); ii) maximum number of mismatches; and iii) distance from the start of oligo/transcript alignment to oligo 5’ end. The rationale for the choice of these parameters is based on data presented in Additional file 8. The mean of all expression values (both control and infected conditions, Additional file 8, panel A) for all 23,284 probes (i.e. no filtering) was 945 (727 when considering only probes with an alignment <= 40%; data not shown). When probes are selected by filtering with the above parameters, the mean of all expression values reaches the maximum for probes filtered for percent alignment = 100% and 0 mismatches (808 probes conserved; mean hybridization value 2,424) (Additional file 8, panel A). On the other hand, when less stringent values were tested, down to the very relaxed >= 30% alignment and up to 3 mismatches, mean expression values drop to about 900 (23,284 probes conserved) (Additional file 8, panel A). This variation in expression values is consistent with the fact that, when the homology between chip oligos and eggplant transcripts is high, high hybridization values are detectable. Additionally, the influence of the distance between the starting point of oligo alignment and the respective Blast hit within 5′ end was monitored (Additional file 8, panels B to E). In fact, oligos are attached to the chip in the 3’ side and this causes steric hindrance in the crowded 3’ regions to interfere with hybridization [30]. Therefore, poor homology along oligo 3’ region against interrogating transcripts can be expected to be less influential on hybridization intensity, as confirmed in our data No effect could be detected when selection was made for distance of alignment from 3’ side (data not shown). We further checked that the variation in mean hybridization is not a mere consequence of the varying number of probes filtered at the different imposed stringencies. A plot where a number of random probes corresponding to the number of probes resulting by setting stringency conditions is shown in Additional file 8F. As expected, no meaningful variation in signal strength is detectable in this case, ruling out that simply the number of probes, irrespective of probe *vs.* interrogating sequence homology, is influential. All these observations are summarized in Additional file 8, where percent alignment, number of mismatches within the alignment and oligo alignment start position are plotted *versus* mean hybridization values and number of retained probes.

**Figure 4:**
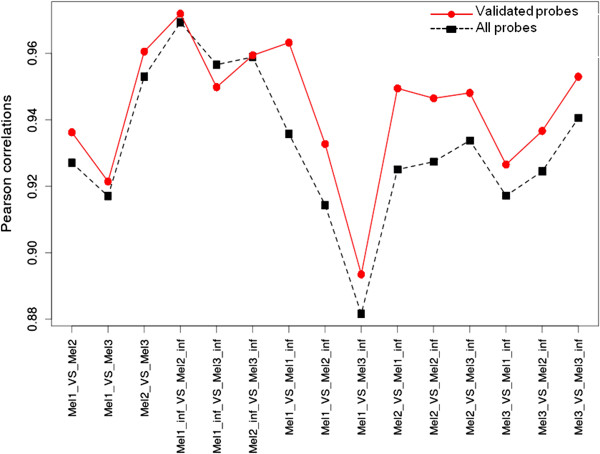
**Pearson correlations among eggplant samples using total vs. validated probes.** Pearson’s product moment correlation for eggplant samples were computed for the entire set of probes or the subset of eggplant validated set of probes. Correlations are shown for all pairs of sample combinations.

Based on the above data, in order to define an high confidence pool of probes for eggplant, we selected a reasonable tradeoff between stringency and number of preserved probes: i) ratio of alignment length to oligo length >= 0.6; ii) maximum number of mismatches <= 2; iii) start of oligo alignment within the first 20 bases from 5’ oligo end. These parameters lead to 5,770 retained oligos with a mean signal of 1,525, a value well above the mean of 727 obtained when only probes with an alignment <= 40% were considered (data not shown) and is still higher than 945, the mean of all probes in the chip (Additional file 8A). To finally verify that our probe validation procedure was beneficial, we computed the Pearson’s product moment correlation values for eggplant samples. The correlation increased in most cases (14 out of 15 pair wise comparisons) when comparing validated *versus* total probes (Table 2 and Figure 4) as expected by removal of probes where, due to the absence of a matching transcript target, random noise prevails leading to inconsistencies among replicates [29].

**Table 2:** Correlation values among eggplant samples prior or after validation

**Validated genes (5770 genes)**						
	**Mel1_ctrl**	**Mel2_ctrl**	**Mel3_ctrl**	**Mel1_infected**	**Mel2_infected**	**Mel3_infected**
**Mel1_ctrl**	1					
**Mel2_ctrl**	0.93623	1				
**Mel3_ctrl**	0.92142	0.96054	1			
**Mel1_infected**	0.96322	0.94946	0.92652	1		
**Mel2_infected**	0.93271	0.94647	0.93664	0.97194	1	
**Mel3_infected**	0.89345	0.94810	0.95296	0.94982	0.95941	1
**ALL GENES (23284)**						
	**Mel1_ctrl**	**Mel2_ctrl**	**Mel3_ctrl**	**Mel1_infected**	**Mel2_infected**	**Mel3_infected**
**Mel1_ctrl**		0.92705	0.91702	0.93579	0.91434	0.88157
**Mel2_ctrl**	0.92705		0.95298	0.92503	0.92737	0.93377
**Mel3_ctrl**	0.91702	0.95298		0.91714	0.92448	0.94053
**Mel1_infected**	0.93579	0.92503	0.91714		0.96931	0.95660
**Mel2_infected**	0.91434	0.92737	0.92448	0.96931		0.95889
**Mel3_infected**	0.88157	0.93377	0.94053	0.95660	0.95889	

Pearson’s product moment correlation values for eggplant samples are compared.

### Overall comparison between Torvum *vs.* eggplant DEGs

As for Torvum samples, eggplant total RNA samples were extracted from control and 14-d inoculated roots (three biological replicates for each condition) and used for array hybridization. In contrast with Torvum, numerous galls and egg-masses were visible in eggplant at the end of the infection process confirming a successful establishment of the infection and reproduction. DEGs calling using the same values as for Torvum (FDR <=0.1 and fold change >=2) resulted in 1,217 genes over the entire 23,284 chip gene set, a value approximately 3-fold larger than found for Torvum genes (Additional file 9). When the 5,770 validated genes were considered, 250 eggplant DEGs were obtained. Thus, 5.2 and 4.3% DEGs were respectively obtained if the validated or total gene sets are chosen. Only 43 and 13 of these eggplant DEG were in common with Torvum DEGs for total and validated dataset, respectively (data not shown).

We further conducted GO enrichment analysis with both the raw and validated eggplant dataset. For validated dataset, prior to Fisher’s test, non-validated genes were subtracted from test and reference sets. No enriched GO terms were revealed using either total or validated dataset.

Eggplant responses to infection at 14 dpi appear to involve modulation of a substantial amount of genes whose large majority is distinct from Torvum DEGs. Furthermore, such modulation appears more sparse and heterogeneous as indicated by a lack of GO enrichment. This scenario is likely attributable to successful infection in eggplant as well as to the late infection stage monitored (14 dpi). In fact, it is expected that wide gene modulation (as distinct from Torvum incompatible interaction) occurs due to the establishment of infection in eggplant. Indeed, compatible interaction involves complex rearrangements as development of multinucleate feeding sites, reallocation of nutritional resource to fulfill pathogen needs, root-galling circuitry and further morpho-physiological alterations to cope with enzyme-rich nematode secretions [31,32]. Indeed, more DEGs were found in compatible *vs.* incompatible interaction of *Meloydogine* spp. in cowpea hosts [28]. The lack of GO enrichment for eggplant DEG as opposed to Torvum DEG may reflect a more targeted response of Torvum genes towards activation of specific defense responses against nematodes.

In the following sections, the heatmaps of expression data where Torvum and eggplant are compared include the information of eggplant validation (probes boxed in red). This allows to evaluate eggplant expression data with additional reliability hints based on the existence in eggplant databases of a transcript with satisfactory homology to Torvum probes. It should be stressed, however, that the absence of a validated probe for eggplant may also indicate that the expression of this gene is Torvum-specific; alternatively, the gene may be expressed at very low levels in eggplant causing its absence in expression repositories. Both of these information may be valuable to pinpoint Torvum-specific expression responses.

### Chitinases

**Figure 5:**
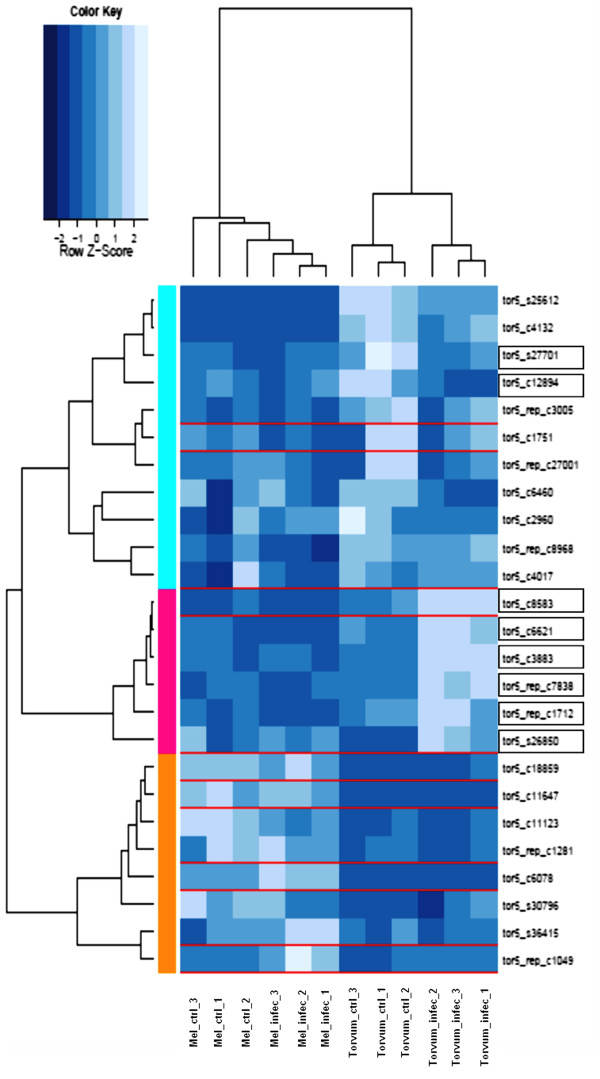
**Heatmap of chitinase transcript abundance.** Torvum and eggplant (torvum and mel prefix, respectively) transcript abundance as affected by *M. incognita* infection. The IDs of modulated Torvum genes (DEG) are boxed in black, while eggplant validated genes are boxed in red over the heatmap. Colored bars on the left of the heatmap mark distinct major branches in the clustering tree grouping genes with similar expression pattern. The color scale indicates the transcript abundance value (light blue indicate higher abundance value, darker blue indicates lower abundance values).

As shown in the bar chart in Figure 2, chitin binding, chitin-catabolic process and chitinase activity are enriched GO terms in Torvum DEGs. Figure 5 depicts an heatmap of the 25 Torvum genes annotated with the GO term ‘chitinase activity’ (GO ID = GO:0004568). Eight of these 25 genes are modulated in Torvum (ID boxed in black). Six of these are induced by infection and cluster together (pink sidebar). Intriguingly, only one probe (namely tor5_C8583, corresponding to class II chitinase) is among eggplant validated probes. This finding, while inviting caution on eggplant expression data for this cluster, indicates that, to date, no other transcripts have been reported for eggplant in this cluster suggesting that these genes might be absent or expressed at very low levels in eggplant. Thus, those chitinases may represent a group of nematode-responsive genes whose presence and/or inducibility recruitment is an exquisite feature of nematode-challenged Torvum.

Chitinases have long been known to be involved in defense responses against nematodes ([33,34] and references therein). In particular, soybean cultivars exhibiting enhanced resistance to *M. incognita* displayed earlier induction and higher overall chitinase activity when compared to susceptible cultivars [35]. Furthermore, chitinases transcripts have been found to be upregulated in transcript profiling experiments of *M. incognita*-challenged cotton roots [20] Although only the egg shell of nematodes contains chitin as a constituent, chitinases may interfere with nematode vitality at various stages of infection [33]. These previous findings have prompted transgenesis approaches aiming at introducing exogenous chitinase in crops. In preliminary attempts with chitinases from *Manduca sexta*[36] and the fungus *T. harzianum*[37], chitinases did not provided enhanced resistance to nematodes when introduced in soybean and tobacco, respectively. This may indicate that native, specific chitinase isoforms and/or appropriate conditions (e.g. tissue-specific expression) as experienced by the native chitinases may be required to exert nematode protection activity. For example, concomitant action of protease may be required to effectively disrupt nematode chitin [38]. More recently, an active chitinase, *PjCHI-1*, has been identified in *Paecilomyces javanicus*, a non-nematophagous fungus. When expressed in tomato, *PjCHI-1* has been shown to reduce egg masses and repress the embryonic development of *M. incognita*[39,40]. *PjCHI-1* belongs to class V of chitinase family 18 [35] and, intriguingly, tor5_c6621 shows as best blast hit tobacco class V chitinase (CAA54373). Details on induced Torvum chitinases are listed in Table 3. Induced chitinases (from 4- to 2-fold) appear to belong to distinct classes, probably reflecting that efficient chitinase activity may require a pool of various chitinase classes acting in concert. Identification at the sequence level of Torvum nematode-induced chitinase may thus help to narrow down the choice of chitinase isoforms to effective ones for introgression into susceptible crops.

**Table 3:** **Blast results of modulated transcripts grouped in GO term ‘chitinase activity**’

**ID**	**Blast2GO annotation**	**Length**	**Best hit desc.**	**Conserved domains**	**Hit ACC**	**E value**	**Expr ratio (infected/control)**
Tor5_s26850	Wound-induced protein win2	443	Gi|19962|emb|CAA41437.1|pathogenesis-related protein 4A [Nicotiana tabacum]	Barwin family pfam 00967	CAA41437	5.87E-036	3.94
Tor5_c3883	Wound-induced protein win1	644	Gi|19962|emb|CAA41437.1|pathogenesis-related protein 4A [Nicotiana tabacum]	Barwin family pfam 00967	CAA41437	6.97E-083	3.96
Tor5_c8583	Class ii chitinase	568	Gi|544010|sp|Q05540.1|CHIB_SOLLC RecName: Full=Acidic 27 kDa endochitinase; Flags: Precursor >gi|19187|emb|CAA78844.1| chitinase [Solanum lycopersicum]	Glycoside hydrolase family 19 chitinase domain	Q05540	7.18E-101	3.49
		
Tor5_c6621	Class v chitinase	694	Gi|899342|emb|CAA54373.1|chitinase class V [Nicotiana tabacum]	Class V plant chitinases Glycosyl hydrolase family 18 Specific hit Cd02879 Superfamily cl10447	CAA54373	1.52E-090	2.55
Tor5_rep_c7838	Class i extracellular chitinase	669	Gi|21495|emb|CAA47921.1|chitinase [Solanum tuberosum]	Specific hit: Glycoside hydrolase family 19 chitinase domain (cd00325) Superfamily: lysozime like domain (cl00222)	CAA47921	2.89E-094	2.24
Tor5_rep_c1712	Chia_tobac ame: full=acidic endochitinase flags: precursor	627	Gi|116332|sp|P29060.1|CHIA_TOBACRecName: Full=Acidic endochitinase; Flags: Precursor >gi|19775|emb|CAA77656.1| acidic chitinase III [Nicotiana tabacum]	Specific hit: cd02877 Superfamily: GH18 Glycosyl hydrolase family 18 Cl10447	P29060	6.82E-098	2.03

### Sesquiterpene biosynthetic enzymes

**Figure 6:**
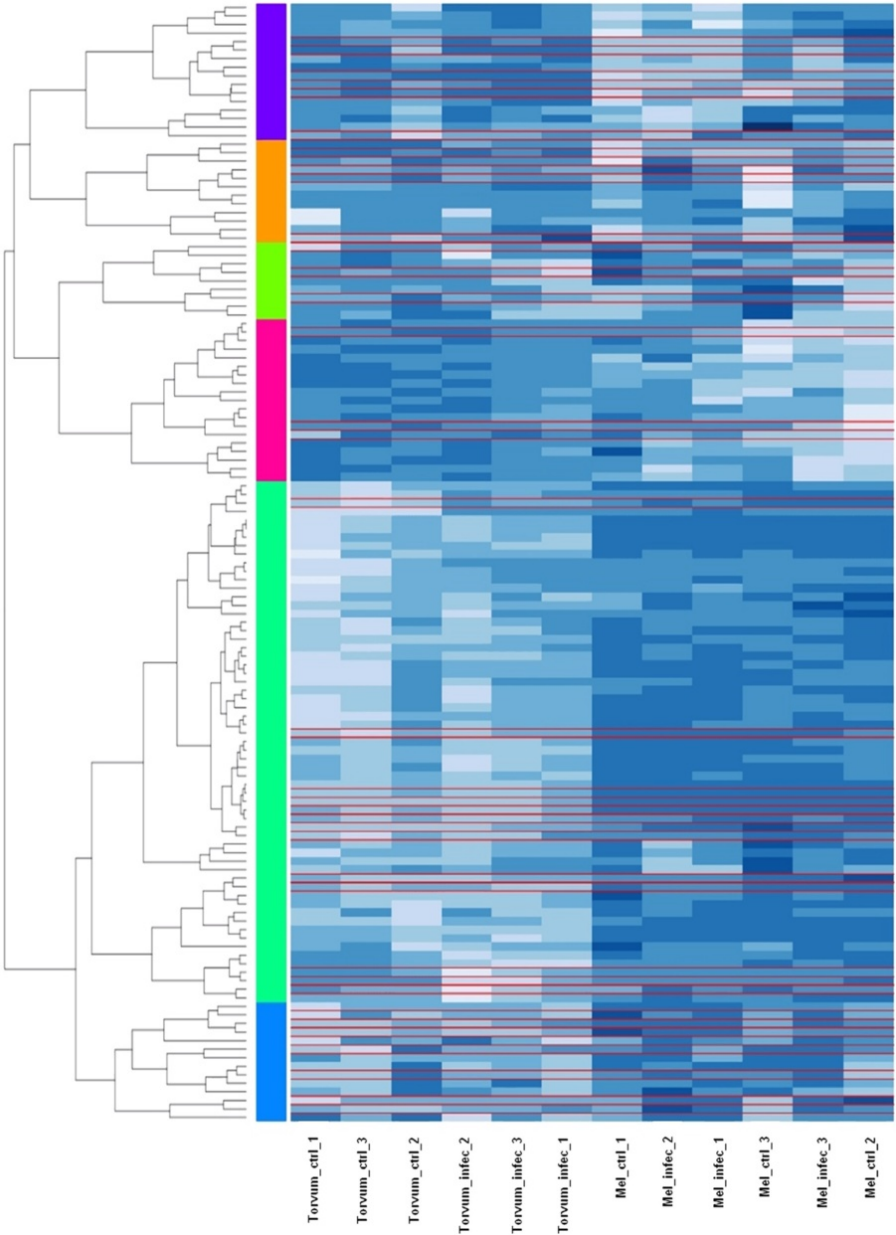
**Heatmap of isoprenoid biosynthetic process transcript abundance.** Overview of transcript abundance in Torvum and eggplant (torvum and mel prefix, respectively) as affected by *M. incognita* infection for the 131 members of GO class ‘isoprenoid biosynthetic process’ term (GO:0008299). Eggplant validated genes are boxed in red over the heatmap. Colored bars on the left of the heatmap mark distinct major branches in the clustering tree grouping genes with similar expression pattern. The color scale indicates the expression value (light blue indicate higher abundance value, darker blue indicates lower abundance values).

**Figure 7:**
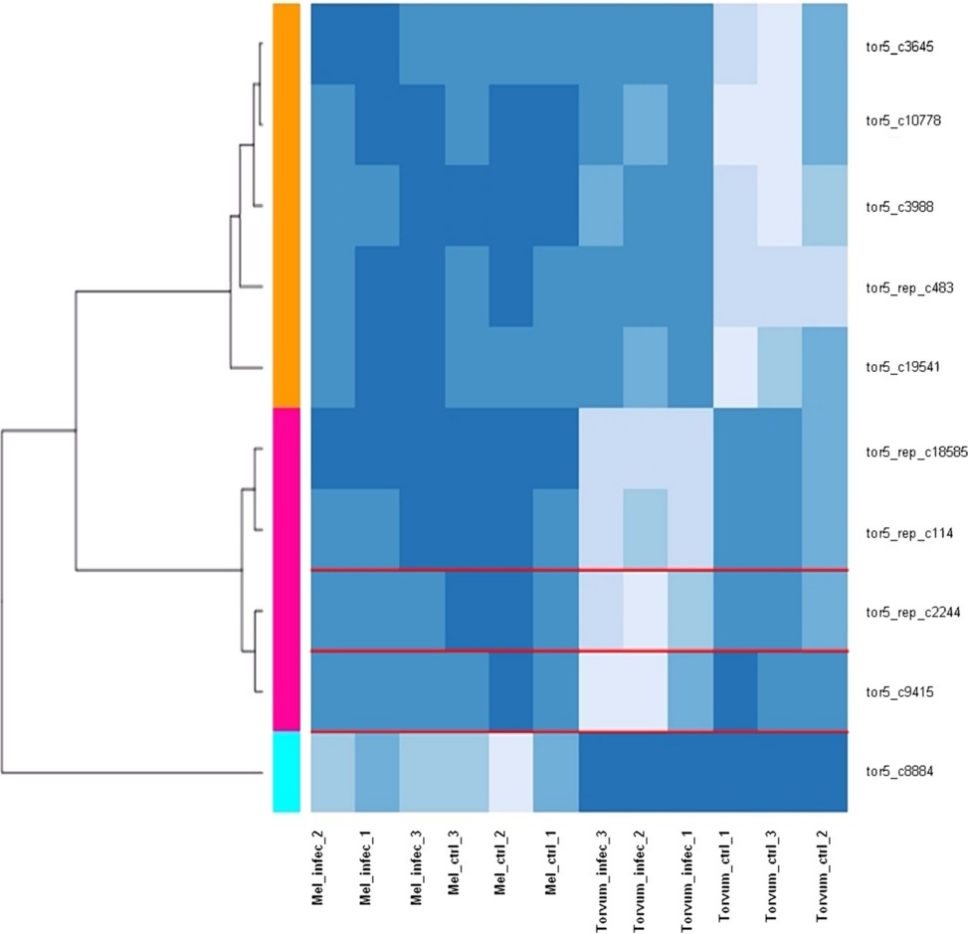
**Heatmap of Torvum DEG (modulated) genes in class isoprenoid biosynthetic process.** Expression patterns of the 10 modulated Torvum genes in GO class ‘isoprenoid biosynthetic process’ term (GO:0008299) are shown. Eggplant validated genes are boxed in red over the heatmap.

GO analysis of torvum DEGs revealed an enrichment for ‘isoprenoid biosynthetic process’ term (GO:0008299). Figure 6 provides an overview of expression pattern of 131 Torvum genes annotated with this term. Noteworthy, Torvum samples cluster together, while eggplant samples (despite several validated genes boxed in red) appear to respond less coherently, as control and infected samples do not cluster together. Figure 7 details expression patterns for the 10 modulated Torvum genes (eggplant validated counterparts boxed in red), while fold change data and the top blast hit for each of the modulated genes are reported in Table 4. Intriguingly, within this group of ‘isoprenoid biosynthetic process’ DEGs, sesquiterpene and diterpenoid biosynthetic genes undergo opposite modulation, i.e. induced the former and repressed the latter. In plants, diterpenes (20C, 4 isoprene units) are produced *via* the plastid pathway (MEP/DOXP pathway), whereas sesquiterpenes (15C, three isoprene units) derive from cytosolic mevalonate pathway [41]. Based on Blast2GO annotation and BlastX hits as reported in Table 5, tor5_c9415 encodes for a cytP450 enzyme with as best hits several CYP450, including epi-aristolochene 1,3 dihydroxylase and premnaspirodiene oxygenase (Cyt P450 71D55) [42]. This P450 enzyme catalyzes several hydroxylations for sesquiterpene substrates including phytoalexins as solavetivone. In turn, tor5_rep_c18585 and tor5_rep_c114 show both as best hit sesquiterpene synthase 2, while tor5_c8884 shows as best hit potato vetispiradiene synthase. The tor5_rep_c2244 (best hit 3-hydroxy-3-methylglutaryl CoAa reductase; HMGR) and tor5_c9415 (best hit cytochrome p450) are the only two genes within Torvum DEGs present in the validated eggplant dataset although they are not differentially expressed. HMGR plays a critical role in isoprenoid biosynthesis as catalyzes the first committed and rate limiting step in isoprenoid biosynthesis [43]. Thus, its presence in validated eggplant is a *bona fide* comparative clue towards the fact that the isoprenoid biosynthetic pathway is not up-regulated (at least at transcriptional level) following nematode infection in eggplant.

**Table 4:** Blast results for modulated isoprenoid biosynthetic process transcripts

**ID**	**Fold change (infected vs ctrl)**	**FDR**	**Blast2GO annotation**	**Hit description**	**Hit E value**	**Hit max identity (%)**	**Hit accession**
Tor5_c9415	5.30	0.000627	Cytochrome p450	Cytochrome P450 71D7 >gb|AAB61965.1| putative cytochrome P450 [Solanum chacoense]	3.00E-061	100.0	P93531.1
Tor5_rep_c18585	2.86	0.001475	Af279456_1 sesquiterpene synthase 2	Terpene synthase [Solanum lycopersicum]	4.0E-022	70.00	AEP82784.1
Tor5_c8884	2.77	0.003101	Vetispiradiene synthase	Vetispiradiene synthase [Solanum tuberosum]	2.0E-116	98.00	BAA82141.1
Tor5_rep_c2244	2.39	0.005414	3-hydroxy-3-methylglutaryl coenzyme a reductase	3-hydroxy-3-methylglutaryl-coenzyme A reductase 2; Short=HMG- CoA reductase 2 >gb|AAD28179.1|AF110383_1 3-hydroxy-3-methylglutaryl-coenzyme A reductase [Capsicum annuum]	1.00E-073	99.00	Q9XEL8.1
Tor5_rep_c114	2.37	0.026802	Af279456_1sesquiterpene synthase 2	Terpene synthase [Solanum lycopersicum]	2.00E-051	73.00	AEP82784.1
Tor5_rep_c483	0.49	0.00577	Ent-Kaurene oxidase	Ent-kaurene oxidase [Pyrus communis]	8.00E-055	75.00	AEK01241.1
Tor5_c10778	0.48	0.038335	Copalyl diphosphate synthase	Copalyl diphosphate synthase [Solanum lycopersicum] >dbj|BAA84918.1| copalyl diphosphate synthase [Solanum lycopersicum]	5.00E-049	72.00	NP_001234008.1
Tor5_c19541	0.44	0.072615	Gibberellin 2-oxidase	Gibberellic acid 2-oxidase [Capsicum annuum]	1.00E-060	84.00	ABE98327.1
Tor5_c3988	0.38	0.057934	Geranylgeranyl diphosphate synthase	Geranylgeranyl pyrophosphate synthase 1 [Solanum pennellii]	3.00E-048	92.00	ADZ24718.1
Tor5_c3645	0.35	0.007791	Cytochrome p450	Uncharacterized protein LOC100790001 precursor [Glycine max] >gb|ACU19728.1| unknown [Glycine max]	7.00E-049	76.00	NP_001242838.1
				Taxane 13-alpha-hydroxylase [Medicago truncatula] >gb|AET00449.1| Taxane 13-alpha-hydroxylase [Medicago truncatula]	3.00E-046	75.00	XP_003617490.1

**Table 5:** **Selected Torvum transcripts annotated as R genes with an expression ratio in Torvum of at least 1.3 (infected *vs *control)**

**ID**	**B2GO sequence description**	**Length**	**Best hit description**	**Eggplant mean expr control**	**Eggplant mean expr infected**	**Torvum mean expr control**	**Torvum mean expr infected**	**Eggplant expr ratio**	**Torvum expr ratio**
Tor5_c12566	Disease resistance identical	710	Gi|54261825|gb|AAV31175.1|Putative disease resistance protein identical [Solanum tuberosum]	168.426	396.871	485.109	654.998	2.356	1.350
Tor5_c14147	Leucine-rich repeat containing	493	Gi|212717155|gb|ACJ37419.1|NBS-LRR disease resistance protein [Glycine max]	1030.461	801.279	370.852	511.059	0.778	1.378
Tor5_c15732	Nbs-lrr resistance protein	498	Gi|224117364|ref|XP_002317554.1|nbs-lrr resistance protein [Populus trichocarpa] >gi|222860619|gb|EEE98166.1| nbs-lrr resistance protein [Populus trichocarpa]	689.272	692.443	258.673	398.725	1.005	1.541
Tor5_c2525	Af202179_1disease resistance protein bs2	664	Gi|6456755|gb|AAF09256.1|AF202179_1disease resistance protein BS2 [Capsicum chacoense]	171.605	69.228	1736.174	2279.920	0.403	1.313
Tor5_c4459	Nbs-lrr type resistance protein	510	Gi|83630761|gb|ABC26878.1|NRC1 [Solanum lycopersicum]	1792.413	1541.649	760.225	1283.284	0.860	1.688
Tor5_c4728	Disease resistance identical disease resistance protein	625	Gi|83630761|gb|ABC26878.1|NRC1 [Solanum lycopersicum]	196.591	152.134	449.926	664.659	0.774	1.477
Tor5_c6199		715	gi|255561552|ref|XP_002521786.1|Disease resistance	233.431	212.023	197.292	304.554	0.908	1.544
			Protein RPP13 putative [Ricinus communis] >gi|223538999|gb|EEF40596.1| Disease resistance protein RPP13. putative [Ricinus communis]						
Tor5_c7069	Nbs-lrr resistance protein	685	Gi|15418712|gb|AAG31015.1|tospovirus resistance protein C [Solanum lycopersicum]	294.835	199.927	811.956	1100.840	0.678	1.356
Tor5_c7274	Disease resistance protein	608	Gi|323370547|gb|ADX43928.1|ADR1 [Solanum tuberosum]	11454.467	12324.265	1568.053	2488.333	1.076	1.587
Tor5_c8985	Disease resistance- dirigent domain-containing protein	490	Gi|224112881|ref|XP_002316318.1|predicted protein [Populus trichocarpa] >gi|222865358|gb|EEF02489.1| predicted protein [Populus trichocarpa]	1862.947	777.762	678.101	992.523	0.417	1.464
Tor5_c9198	Nbs-lrr resistance protein	672	Gi|3426261|gb|AAC32253.1|disease resistance gene homolog Mi-copy1 [Solanum lycopersicum]	360.807	155.364	188.721	282.809	0.431	1.499
Tor5_rep_c1655	Disease resistance response protein	803	Gi|224105209|ref|XP_002313728.1|predicted protein [Populus trichocarpa] >gi|222850136|gb|EEE87683.1| predicted protein [Populus trichocarpa]	77.749	138.308	345.620	512.257	1.779	1.482
Tor5_rep_c4425	Nbs-lrr resistance protein	574	Gi|15418710|gb|AAG31014.1|tospovirus resistance protein B [Solanum lycopersicum]	224.075	149.135	339.977	456.547	0.666	1.343
Tor5_rep_c7021	Disease resistance protein	519	Gi|53749455|gb|AAU90310.1|hypothetical protein STB1_54t00008 [Solanum tuberosum]	252.559	282.853	229.561	303.609	1.120	1.323
Tor5_rep_c9578	Disease resistance protein	472	Gi|53749455|gb|AAU90310.1|hypothetical protein STB1_54t00008 [Solanum tuberosum]	470.054	344.881	274.840	413.509	0.734	1.505
Tor5_s25440	Disease resistance protein	473	Gi|224117364|ref|XP_002317554.1|nbs-lrr resistance protein [Populus trichocarpa] >gi|222860619|gb|EEE98166.1| nbs-lrr resistance protein [Populus trichocarpa]	10260.362	8458.349	1366.653	3326.171	0.824	2.434

While an in-depth understanding of sesquiterpenoids biochemical reactions undergoing in nematode-challenged Torvum will require obtaining full-length transcripts from the Torvum transcripts, which in our dataset are limited by design to the 3’ region, the fact that the biosynthetic sesquiterpenoid pathway is stimulated points to sesquiterpenoids as critical effectors of Torvum resistance mechanisms. Sesquiterpenoids include several compounds of well-established nematotoxic and nematostatic effects as gossypol, solavetivone, rishitin and lubumin [44], and references therein. Gossypol is a very effective sesquiterpene aldehyde shown to cause resistance to *Meloydogine incognita*[45,46] in cotton and solavetivone, rishitin and lubumin are bicyclic sesquiterpenoids expressed in solanaceous species as tomato and potato capable of causing nematostatic effects [31,33]. High content of solavetivone (associated to the H1 resistance gene) was found to be critical for conferring resistance to *Globodera rostochiensis*[47]. Rishitin was shown to be induced by plant-parasitic nematodes and higher rishitin contents in various potato species were associated to higher nematode resistance. In particular, the action of rishitin or of a rishitin-like compound causing nematode to migrate away from plants, would be consistent with observed the poor establishment on parasites on Torvum plants [48].

It should be noted, however, that a substantial number of plant phytochemicals exhibit antinematodal activity and that evidence on the antinematodal activity of sesquiterpenoids is largely based on *in vitro* studies [31,33,44,45]. Few studies monitored the accumulation of sesquiterpenoids upon elicitation and, in only a subset of them, as in cotton roots [45], elicitation was driven by *M. incognita* infection, more frequently, general and unspecific elicitors as arachidonic acid were employed. A further work based on six cotton cultivars, did not support a correlation between resistance and sesquiterpenoid content [46]. Nevertheless, it should be noted that in the above mentioned elicitation studies, extraction and detection techniques were tailored to reveal a narrow range of compounds as low-molecular weight, non-polar chemicals [45,47]. This approach prevents and unbiased, global overview of the whole spectrum of defense strategies mounted by the host to counteract infection, and thus hampers an assessment of their relative contribution to actual resistance.

Prior to our report, no large-scale transcriptional profiling of *M. incognita*-infected resistant *vs*. susceptible genotypes, had revealed transcriptional induction of sesquiterpenoid genes [17,21]. This fact may be attributable to the limited number of genes tested in previous transcriptional profiling studies, and/or to further factors as genotypes under investigation and sampling time. Transcriptional induction of sesquiterpenoid genes associated to the resistant genotype Torvum, but not to the susceptible counterpart eggplant, strongly points to sesquiterpenoids as key effectors of Torvum resistance against *M. incognita* and sheds lights on their activation mode and relative contribution to the battery of Torvum defense strategies.

Overall, it appears that Torvum biosynthetic resources are channeled towards sesquiterpene biosynthesis at the expense of distinct biosynthetic branches. Further work based on the Torvum sequences reported here will likely provide the tools for an in-depth understanding of Torvum responses ultimately pointing to key phytoalexin products.

### Identification of Torvum resistance gene analogs

Several resistance genes against plant-parasitic nematodes have been cloned [12]. Among them the best-studied is the tomato gene *Mi*, which exhibits a broad resistance pattern to root-knot nematodes as well as to phloem-feeding insects, including *Macrosiphum euphorbiae* (potato aphid) and to the white fly *Bemisia tabaci*. Further nematode R genes cloned are: *Hs1pro-1*, *Hero*, *Gpa2*, *Gro1-4*, *Rhg1* and *Rhg4R*[12].

Several aspects of the interaction among Torvum and *Meloydogin*e would be consistent with an early rejection mechanism as ensured by vertical resistance (i.e. mediated by Avirulence-Resistance cognate gene interaction). In the Additional file 10 we report the 81 Torvum transcripts with annotations referring to candidate R genes analogs (RGAs). Among these, 28 (34%) and 47 (58%) showed enhanced expression in infected *vs.* control samples, respectively, in eggplant and Torvum. While no transcriptional modulation is strictly required for a R gene to be considered a candidate R gene (transcription of many R genes is not responsive to pathogen infection) several active R genes show moderate level of pathogen responsiveness in terms of transcription (e.g. 30% induction or more over controls), e.g. *Xa1, Xa2*7 [49,50]. Thus, monitoring the expression patterns of Torvum candidate R genes analogs (RGAs) can help pointing to RGAs of interest [51]. Table 5 enlists 16 Torvum transcripts annotated as disease resistance genes showing an expression ratio (infected vs. control) of at least 1.3. Only one of these Torvum induced transcripts has a validated counterpart in eggplant (tor5_c4459, slightly downregulated as a consequence of infection) pointing to major sequence divergence and/or lack of counterparts in eggplants for this set of induced Torvum candidate resistance genes. Intriguingly, among the candidate induced RGAs one shows as best hit a homologous to *Mi* nematode resistance gene (tor5_c9198).

**Figure 8:**
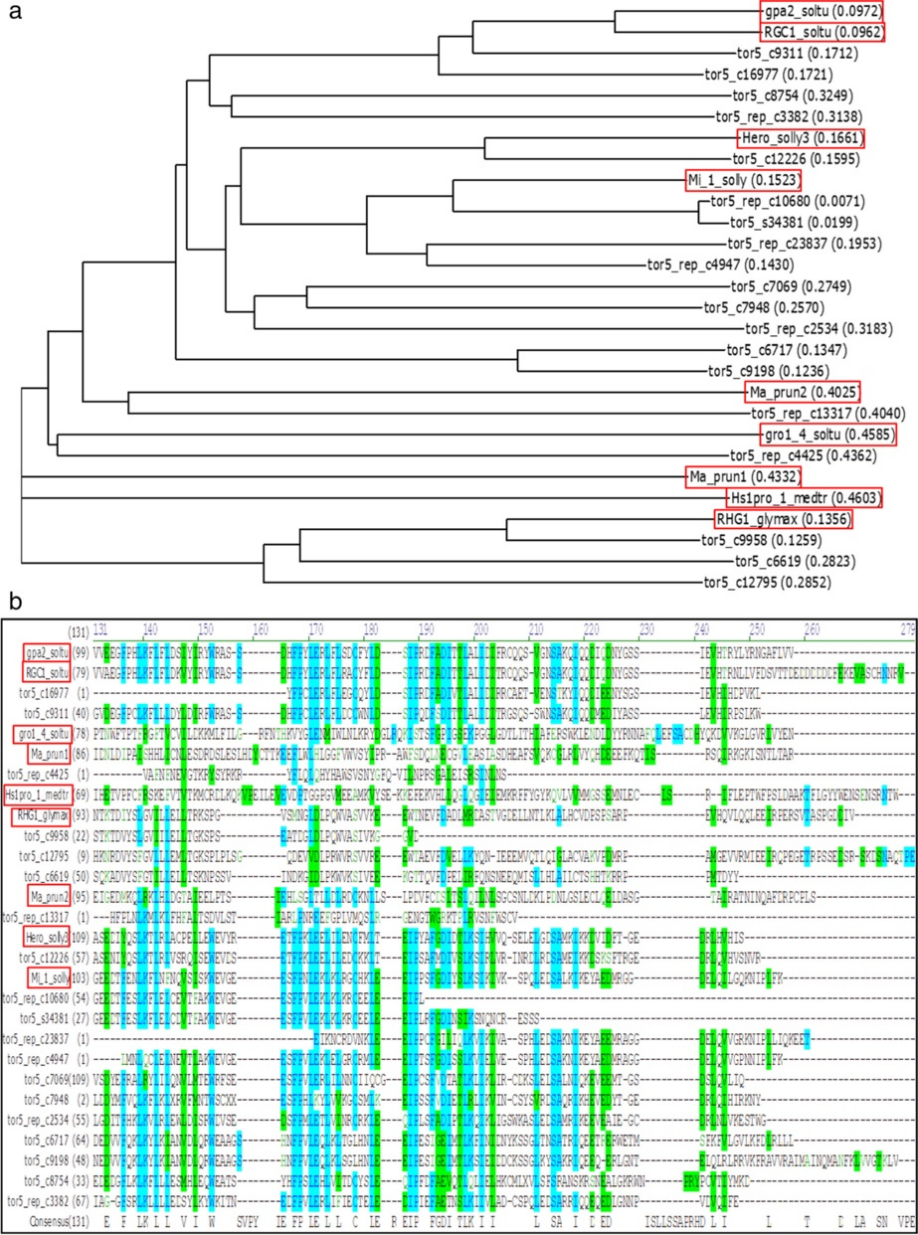
**Multiple alignment of selected Torvum translated transcripts to known nematode resistance gene proteins.** The longest ORF of Torvum transcripts were aligned to the most C-terminal residues of a selection of well characterized (boxed in red) plant nematode R genes. **a)** dendrogram. **b)** sequence alignment detail. Plant nematode R genes: Gpa2_soltu=disease resistance protein Gpa2 *Solanum tuberosum* (AAF04603.1); RGC1_soltu= RGC1 *Solanum tuberosum* (AAF76163.1); Hero_solly3=Hero resistance protein 3 homologue *Solanum lycopersicum* (CAD29727.1); Mi_1_solly=plant resistance protein *Solanum lycopersicum* (NP_001234622.1); Ma_prun2=, nematode resistance-like protein *Prunus cerasifera* (CAR94516.1); Gro1_4_solu=nematode resistance protein *Solanum tuberosum* (AAP44390.1); Ma_prun1=nematode resistance-like protein *Prunus cerasifera* (CAR94516.1); Hs1pro_1_medtr=nematode resistance HS1pro1 protein *Medicago truncatula* (AES99551.1); RHG1_glymax=, receptor-like kinase RHG1 *Glycine max* (NP_001235765.1).

Figure 8 depicts a multiple alignment and associated dendrogram encompassing selected Torvum RGAs and the most C-terminal 200 residues of known R genes (boxed in red). Protein alignment was made for Torvum RGAs by selecting the longest ORF. As expected, Torvum sequences by design align to the most C-terminal regions of R genes (where the highly variable LRR region is placed) and only the most C-terminal 100–150 AA of alignments are shown. Despite the fact the aligned regions are the poorly conserved LRR (Leucine Rich Repeat) regions, several Torvum transcripts show homology and cluster close to distinct R-prototype resistance genes and appear good candidates for future assessment of their role as true R genes.

### qPCR validation of selected genes

In order to validate our microarray expression results, we choose six sequences (Table 6) among both upregulated and downregulated DEG of particular relevance as discussed above. Overall, despite the correlation among qPCR and array data (0,704) was non-significant according to Pearson’s product–moment correlation (p=0,118, n=6), the direction of change in expression of qPCR and microarray was in agreement for all the tested transcripts (6 of 6).

**Table 6:** qPCR validation of microarray results

**ID**	**Function**	**Fold change qPCR**	**Exp. trend qPCR**	**Fold change chip**	**Exp. trend chip**	**Consistency among qPCR and chip**
Tor5_c3883	Wound-ind. Prot win1	1.88	+	3.86	+	YES
Tor5_rep_c1712	Acidic endochitinase	1.24	+	2.12	+	YES
Tor5_c8583	Class2 chitinase	5.22	+	3.44	+	YES
Tor5_c6621	Class v chitinase	3.37	+	2.56	+	YES
Tor5_rep_c483	Ent-kaurene ox	0.66	-	0.49	-	YES
Tor5_c3988	Ggdp synth.	0.51	-	0.39	-	YES

## Conclusions

We conducted a cost-effective global transcriptome profiling in *Solanum torvum*, a non-model species, by combining NGS pyrosequencing and microarray technology. As a first step, we generated a 3’ transcript catalogue for Torvum by assembling 500–600 bp reads from a normalized library. By limiting the sequencing to the 3’ region we improved average coverage while conserving specificity (as less conserved 3’ UTR regions are covered). This catalogue represents a substantial advancement along characterization of Torvum transcriptome, since even at the relaxed stringency of an 10^-6^ Expect value more than 60% of Torvum unigenes in our catalogue do not have Blast hits in available Torvum databases. The catalogue was subsequently used to design a custom chip for profiling transcriptome changes as a consequence of nematode infection in nematode-resistant species Torvum and the related nematode-susceptible species eggplant. To assess expression results for the heterologous hybridization conducted with eggplant samples, we developed an *in silico* validation strategy which can be applied in general to extend the use of species-specific chips to samples from phylogenetically related species devoid of a chip. This allows to define, for such heterologous hybridizations, pool of genes whose hybridization data are expected to be more reliable. Finally, the expression profiling of Torvum responses to nematode infection reveals a focused upregulation of various classes of chitinases and, for the first time, of sesquiterpenoids biosynthetic genes and. On the other hand, no such responses are detectable in eggplant, where a large but sparse and incoherent gene modulation occurs, probably as a consequence of successful establishment of infection. The availability of the long sequence tags in Torvum catalogue will allow precise identification of active nematocide/nematostatic compounds and associated enzymes posing the basis for the exploitation of these resistance mechanisms in other species.

## Methods

### Plant materials and growth conditions

Seeds of *Solanum torvum* Sw accession TG1 [1] and eggplant (*Solanum melongena* L.) breeding line 1F5(9) were sown in a seed-plot for germination. Seedlings about 10 cm tall at the second-leaf stage were singly transplanted into 10 cm diameter plastic pots, each containing 500 cc of mixture sterilized sandy soil: 70% sand+ 15% leca + 10% clay (sterilized at 70°C for 24 hours) + 5% organic matter. The pots were maintained in controlled chambers at 24±2°C, 60% relative humidity, with a 16-h light/8-h dark regimen. Plants were watered with tap water at necessity and fertilized every two weeks with a 20-20-20 NPK fertilizer.

### Nematode culture and inoculation

Root-knot nematode (RKN), *Meloidogyne incognita*, was maintained in the greenhouse on tobacco (*Nicotiana tabacum* L.*)* plants [52], egg masses were extracted with 0.5% (v/v) NaOCl and second stage larvae (J2) hatched as described by [53]. Plants were inoculated with about 250–300 J2 and eggs pouring into three holes in the soil just around the base of the plant stem. Pots were maintained at same conditions, as previously indicated, and checked periodically. Treatments and controls were replicated five times. Inoculated roots were observed for galling and egg-masses development two months later inoculation. For transcriptome study, Torvum control and infected plants at 14 days post inoculation (dpi) were produced. Root tissues collected from each type of plant were then used for RNA extractions and microarray analysis.

### Nematode staining and infection assessment

Roots were removed from soil and washed in water for few minutes. Then, each root system was dipped in a 0,15 g/l phloxine B solution for 15 minutes to point out galls and egg masses. Stained roots were observed under the microscope to assess nematode infectivity by estimating the root-galling index [54] and egg masses [55] on a 0–5 scale: root-gall index (0: no gall; 1: 1–5 small galls; 2:<25% roots with galls; 3: 25-50%; 4: 50-75%; 5:>75% roots with galls); egg mass or egg-laying females (0: no egg mass; 1: 1–2 egg masses; 2: 3–10 egg masses; 3: 11–30 egg masses; 4: 31–100 egg masses; 5: >100 egg masses). Nematode infectivity tests were also conducted by employing highly susceptible plants such as *S. melongena*.

### ***RNA extraction***

RNA samples were extracted, as total RNA, from mock-inoculated and infected Torvum and eggplant roots (three biological replicates for each condition) by means of the Nucleospin RNA plant Kit (MACHEREY-NAGEL GmbH & Co., Düren, Germany) at 0 and 14 dpi. Total RNA quality was assessed using a Bioanalyzer 2100 Expert (B.02.07.SI532–, Agilent Technologies, Inc). In all the samples tested, RIN (RNA Integrity number) resulted to be above 9, while the concentration ranged among 100 and 120 ng/μl.

### ***qRT-PCR experiments***

Real time PCR analysis were carried out in a Applied Biosystems 7500HT Fast Real Time PCR System. The 20 μl reaction mixture consisted of 10 μl BIORAD iTaq™ universal SYBR^®^ Green supermix, 2 μl of sample cDNA, 200 nM forward and reverse primers and nuclease-free water. The reference genes used were *Glutatione peroxidase* and *Diaminopimelate carboxilase*; they were chosen among a list of the best performing housekeeping genes, since their expression was uniform in all samples preliminarily tested. Prior to perform correlation analyses, the data were tested for normality using the Shapiro-Francia test. The data were normally distributed and Pearson’s correlation was used.

### ***Custom chip design***

Total RNA was extracted from Torvum tissues (roots and young leaves) grown in a wide range of conditions to allow for ample gene transcription. Such treatments included low temperatures (4°C; 30 minutes), high temperatures (over 30 °C with peaks of 40°C as reached in a non-cooled glasshouse during the whole day for a 4 days), soil-borne fungus (*Verticillium dahliae*) and nematodes (*Meloidogyne incognita*) with sampling at 1, 7 and 14 dpi, wounding (punching holes with a needle, harvest after 1 and 7 days) and drought stress (harvest after 14 days without water supply) RNA samples were pooled and, from 500 ng of total RNA a -3’ cDNA library (500–600 bp) was generated (Eurofins MWG operon) with oligo(dT) primer (first-strand) and random priming (second-strand) and subsequently normalized.

*De novo* assembly of Torvum reads was undertaken with MIRA 3.0.5 [22] in *de novo* assembly mode and conducted with 454-specific parameters.

RNA labelling and hybridizations of the Custom 90K CombiMatrix array were as detailed in Bellin et al., [5]. Gene-specific oligonucleotides were designed with OligoArray 2.1 software [25]. Oligoarray parameters were tuned to for the observed GC content of 38.23% for the unigenes (e.g. Tm 79–86; GC content range 34–60). The final number of probes in the chip was reduced to 30,000, by excluding less specific probes (probes hybridizing with more than one target), in order to allow a triplicate probe layout in the 90k-features Combimatrix gene chip. The final layout consisted in 24,394 probes representative of contigs and 5,606 probes derived from singletons.

### ***Miscellaneous bioinformatic techniques***

For Blast2GO annotation of Torvum catalogue, the 23,284 unigenes included in the chip layout for which an hybridization signal could be obtained were blasted (BlastX) against NCBI non-redundant database. Expect value was set to 1.0E^-6^, HSP length cutoff was 100 and up to 20 hits were allowed to be retrieved. Annotation was according to default parameters with GO weight set to 5 with an annotation cutoff of 30 and minimum HSP of 10. Call of differentially expressed genes (DEG) was performed with R package LIMMA (Linear Models Microarray Analysis) [56] and custom functions. Genes were considered differentially expressed if exhibiting at least a 2-fold change and a False Discovery Rate (FDR) <= 0.1. For chip extension technique eggplant entries from NCBI EST division (98,089 entries as July 2012), and eggplant tentative consensus from various expression databases including DFCI eggplant gene index [57] PlantGDB PUT [58] and SolEST [59] were combined and queried with chip probe sequences using local BlastN at a relaxed stringency (Expect value 10). The Blast output was subsequently parsed by custom scripts to filter probes based on alignment parameters expected to influence hybridization strength. Heatmaps was generated with custom scripts based on heatmap.2 function as available in the ‘gplots’ Bioconductor package. Multiple alignments were generated with AlignX module of Vector NTI. Unless otherwise stated, other graphical outputs were generated with custom R (version 2.15 ) and Python scripts.

### Availability of data

Accessions for dataset are the following: Sequence Read Archive (SRA; http://www.ncbi.nlm.nih.gov/Traces/sra/): SRA experiment: SRX268284; SRA run: SRR830592; Transcriptome shotgun assembly (TSA; http://www.ncbi.nlm.nih.gov/genbank/tsa): GAIC00000000; gene expression omnibus (GEO; http://www.ncbi.nlm.nih.gov/geo/): data have been submitted and are under NCBI processing.

## Abbreviations

Torvum: *Solanum Torvum*; RGAs: Resistance gene analogs.

## Competing interests

The authors declare that they have no competing interests.

## Authors’ contributions

PB carried bioinformatic procedures and drafted the manuscript. TS prepared RNA samples. TI and CS grew Torvum and eggplant plants and conducted nematode inoculations and scored results. AL participated in annotation analysis. MB conducted Real-Time validations. GLR, LC and SS participated in studying, design and analysis and participated in drafting the manuscript. ES conceived the study, analyzed Real-Time validation data and participated in drafting the manuscript. All authors read and approved the final manuscript.

## Supplementary Material

Additional file 1 ***De novo *assembly parameters.** (a) Distribution of contig length in raw assembly output and final chip layout. (b) The distribution of average contig coverage (rounded to an integer value) in raw assembly output. Contigs are grouped based on bins as detailed on x axis. Singletons are displayed as contigs with read coverage equal to one. Click here for file

Additional file 2 **Specificity of Torvum probes designed for custom chip.** The specificity or probes designed by oligoarray over the raw set of Torvum unigenes is shown. Probes strictly specific for only one Torvum unigene have only one associated target. Click here for file

Additional file 3 Pearson correlation coefficients. Click here for file

Additional file 4 Torvum DEG. Click here for file

Additional file 5 Torvum unigene annotations. Click here for file

Additional file 6 Torvum unigene sequences. Click here for file

Additional file 7 Enriched GO terms. Click here for file

Additional file 8 **Overall eggplant expression values as influenced by homology among Torvum probes and eggplant transcripts.** Effect on mean expression values (left side of Y axis ) and number of retained probes (right side of Y axis, reddish bars) as influenced by percent alignment (X axis) and number of mismatches ( 0, 1 or 2 labels in lines). Panel (a) to (e): the effect of imposing an oligo alignment start (from 5’ side) of none, 20, 10, 5 and 1, respectively, is shown. Panel (f): effect of different number of randomly chosen probes on expression values. Click here for file

Additional file 9 Eggplant DEGs. Click here for file

Additional file 10 Torvum RGAs. Click here for file
