# Supplementary figures and images for: Solanum torvum responses to the root-knot nematode Meloidogyne incognita
Source: BMC Genomics. 2013 Aug 9;14:540. doi: 10.1186/1471-2164-14-540 (PMC3750854; doi:10.1186/1471-2164-14-540)

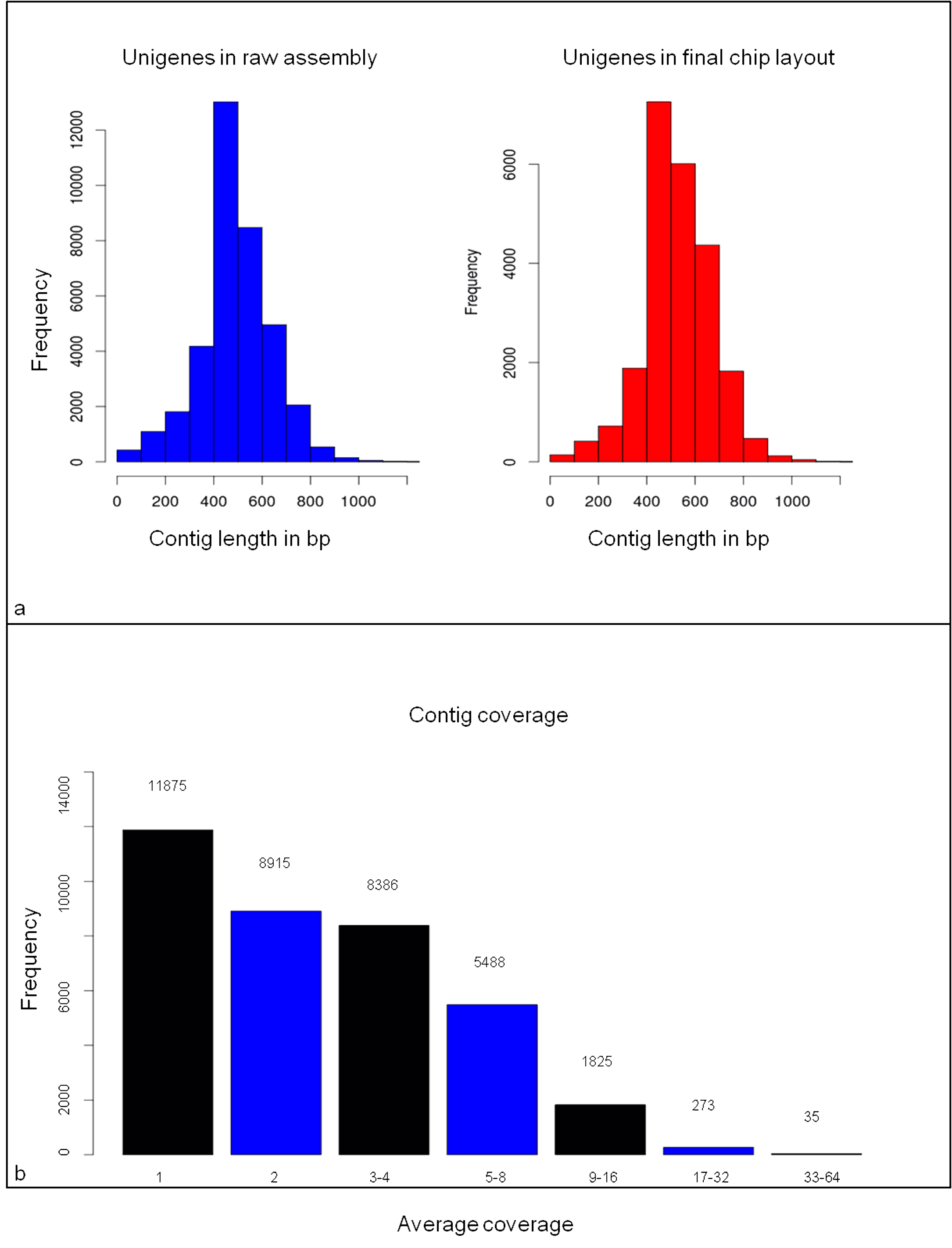

Supplement: Additional file 1 — De novo assembly parameters. (a) Distribution of contig length in raw assembly output and final chip layout. (b) The distribution of average contig coverage (rounded to an integer value) in raw assembly output. Contigs are grouped based on bins as detailed on x axis. Singletons are displayed as contigs with read coverage equal to one. [file 1471-2164-14-540-S1.png]

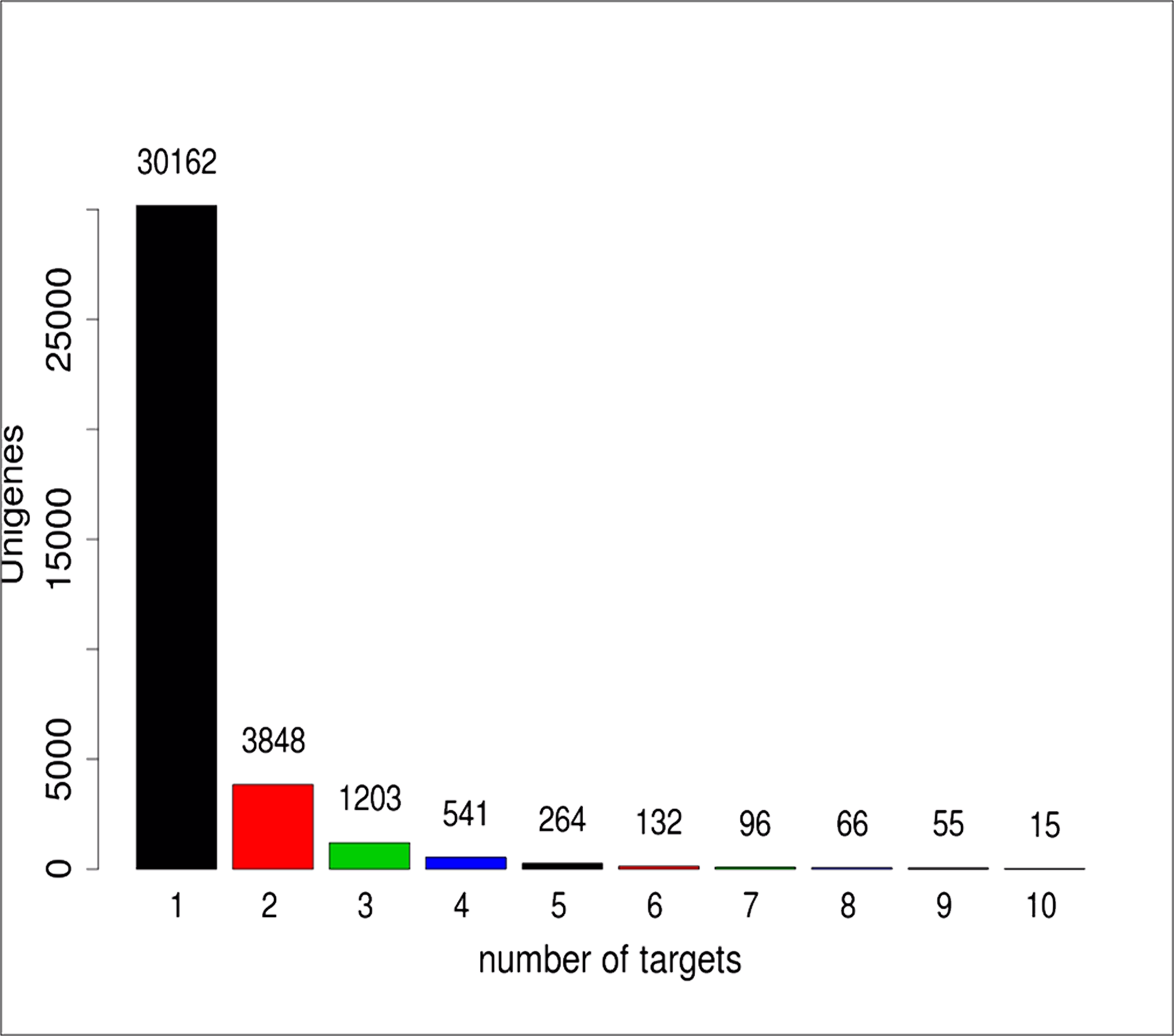

Supplement: Additional file 2 — Specificity of Torvum probes designed for custom chip. The specificity or probes designed by oligoarray over the raw set of Torvum unigenes is shown. Probes strictly specific for only one Torvum unigene have only one associated target. [file 1471-2164-14-540-S2.png]

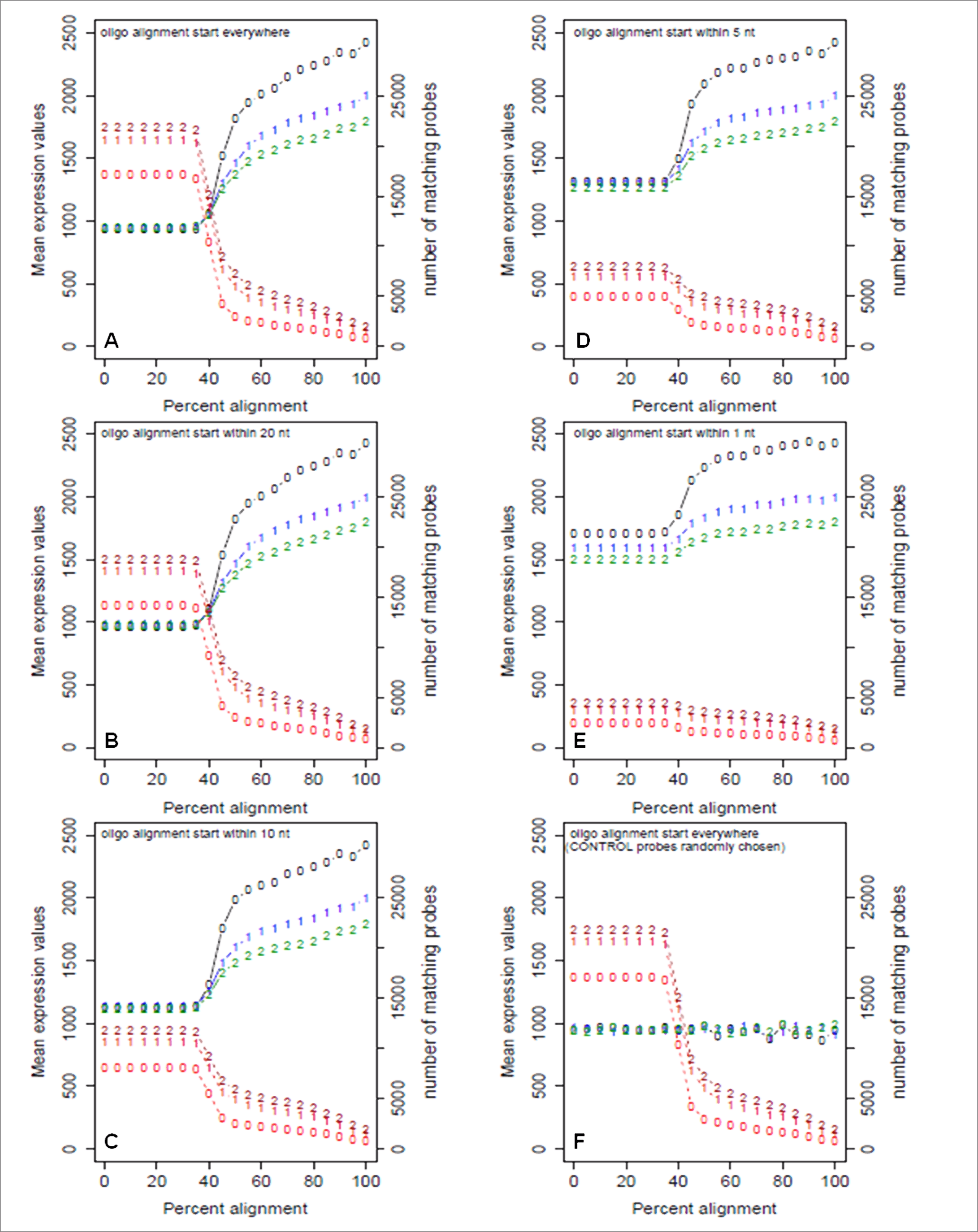

Supplement: Additional file 8 — Overall eggplant expression values as influenced by homology among Torvum probes and eggplant transcripts. Effect on mean expression values (left side of Y axis ) and number of retained probes (right side of Y axis, reddish bars) as influenced by percent alignment (X axis) and number of mismatches ( 0, 1 or 2 labels in lines). Panel (a) to (e): the effect of imposing an oligo alignment start (from 5’ side) of none, 20, 10, 5 and 1, respectively, is shown. Panel (f): effect of different number of randomly chosen probes on expression values. [file 1471-2164-14-540-S8.png]
